# Small Biopsy Samples: Are They Representative for Biphenotypic Sinonasal Sarcoma?

**DOI:** 10.3390/diagnostics12102528

**Published:** 2022-10-18

**Authors:** Olga Kuczkiewicz-Siemion, Monika Prochorec-Sobieszek, Maciej Rysz, Aneta Wojnowska, Monika Durzyńska

**Affiliations:** 1 Department of Pathology, Maria Sklodowska-Curie National Research Institute of Oncology, 02-781 Warsaw, Poland; 2 Reconstructive Team, Department of Breast Cancer and Reconstructive Surgery, Maria Sklodowska-Curie National Research Institute of Oncology, 02-781 Warsaw, Poland

**Keywords:** biphenotypic sinonasal sarcoma, PAX3, BSNS, differential diagnosis, immunohistochemistry, sinonasal tract

## Abstract

(1) Background: Biphenotypic sinonasal sarcoma (BSNS) is a rare low-grade neoplasm of the sinonasal tract. It is characterized by specific *PAX3* gene rearrangements and both myogenic and neural differentiation. The purpose of the study was to describe the histologic, immunohistochemical and molecular features of BSNS and indicate important clues for small incisional biopsy diagnostics. (2) Methods: Archival samples from patients with nasal cavities or ethmoid sinuses tumors were searched for BSNS cases. Inclusion criteria were the presence of spindle cell morphology and low-grade appearance. Both biopsy and resection specimens were stained for identical IHC panels including, i.a., S100, SMA, SOX10 and PAX3. FISH for *PAX3* and *SS18* was performed on biopsy specimens. (3) Results: BSNS diagnosis was made in 6 cases included in the study and confirmed by *PAX3* rearrangement by FISH in 5 specimens. The pattern of IHC expression was identical for paired biopsy and resection samples apart from one BSNS case. (4) Conclusions: Incisional biopsy seems to be a sufficient method to establish BSNS diagnosis in most cases. Characteristic morphological features together with S100, SOX10 and SMA as the screening markers are useful for confirming the diagnosis. In cases of divergent morphology and immunoprofile evaluation of *PAX3* rearrangement is vital.

## 1. Introduction

Biphenotypic sinonasal sarcoma (BSNS) is a rare mesenchymal tumor with little over 100 cases reported so far [1]. This neoplasm was first described by Lewis et al. as a “low-grade sinonasal sarcoma with neural and myogenic differentiation” [2]. The molecular hallmark of BSNS is a rearrangement involving the *PAX3* gene, with *MAML3* being the most prevalent fusion partner [3]. However, diagnostic techniques for the detection of this alteration are not widely accessible.

BSNS is a locally aggressive tumor that arises exclusively in the sinonasal region and presents distinct histologic features [4]. It is characterized by highly cellular, monotonous, bland-appearing spindle cell proliferation, arranged in medium to long fascicles often with prominent herringbone architecture. The tumor presents absence or very low mitotic activity. Tumor necrosis and significant nuclear pleomorphism are exceedingly rare findings [2,5]. Despite the defined histological description, morphology is often not enough to establish the final diagnosis, as similar presentations could be manifested by various neoplasms located in the sinonasal tract. Thus, performing a series of ancillary tests in incisional biopsy specimens seems to be crucial for the diagnosis [6].

Immunohistochemically BSNS expresses both neural crest and myogenic differentiation markers, which can be demonstrated by the positivity of S100 and smooth muscle actin (SMA) stainings. Interestingly, another neuronal marker, SOX10 (SRY-related HMG-box 10) is consistently negative in this tumor type. The intensity and extent of S100 and SMA stainings range from patchy to diffuse patterns, which could be challenging in interpretation, especially in small biopsy samples [2,6].

An immense variety of tumor types may arise in the sinonasal tract and correct diagnosis is vital for providing further management decisions [7,8,9]. BSNS is a locally advanced disease treated by local excision, with or without adjuvant radiation treatment. It has a high local recurrence rate; however, no metastases have yet been reported [6]. BSNS differential diagnoses include other tumors of a low-grade spindle cell morphology, both, with a benign clinical course such as schwannomas and aggressively behaving neoplasms such as synovial sarcomas (SS) [1,9,10].

Due to the morphologic and immunophenotypic diversity of sinonasal tumors, diagnostic decisions could pose a lot of difficulties, especially in the lack of available tissue material [5,11]. This requires a pathologist to follow a specific diagnostic scheme and create a hierarchy of immunohistochemistry evaluation to achieve the final diagnosis with limited material. 

We sought to address multiple aims in this study: first, to describe the histologic, immunohistochemical and molecular features of this rare tumor; second, to validate previously reported IHC findings in the context of differential diagnosis, and, finally, to indicate important clues for small incisional biopsy diagnostics. 

## 2. Materials and Methods

### 2.1. Case Selection

All consecutive patients with nasal cavity or ethmoid sinuses tumors resected surgically from January 2009 to August 2021 in the Maria Sklodowska-Curie National Research Institute of Oncology in Warsaw, Poland were retrospectively analyzed. Our clinical cohort data were searched for mesenchymal neoplasms in the sinonasal tract. Hematoxylin and eosin-stained slides, whenever tissue material was available, were re-evaluated by the pathologist experienced in head and neck pathology (M.D.) and searched for potential BSNS cases. Inclusion criteria were the presence of spindle cell morphology and low-grade appearance. Exclusion criteria were the presence of high-grade atypia, necrosis or atypical mitosis. Cases of osteosarcomas, chondrosarcomas as well as epithelioid cell sarcomas were excluded. Specimens containing bone were initially decalcified in 20% ethylenediaminetetraacetic acid (EDTA) solution at 4 °C with intermittent shaking. Clinical characteristics were obtained from pathology reports and the electronic medical record. The study was performed according to the Declaration of Helsinki. All procedures performed in this retrospective analysis involving human participants were approved by the Institutional Ethics Review Board (reference number: 4/2020).

### 2.2. Immunohistochemistry

Both biopsy and resection formalin-fixed and paraffin-embedded (FFPE) specimens were stained for S100, SMA, SOX10, desmin, h-caldesmon, MyoD1 (myoblast determination protein 1) or myogenin, STAT6 (signal transducer and activator of transcription 6), CD34 (cluster of differentiation 34), CKAE1\3 (cytokeratin AE1/AE3), EMA (epithelial membrane antigen), TLE1 (transducin-like enhancer of split 1), H3K27me3 (trimethylation at lysine 27 of histone H3) and PAX3 (paired box gene 3). The specifications of immunohistochemical stains which were used are presented in Table 1. Expression of PAX3 (dilution 1:300) by IHC was interpreted as positive only when diffuse strong nuclear staining occurred and the expression was distinctly stronger than the surrounding background. 

### 2.3. FISH

The 5-μm-thick FFPE tissue sections were analyzed by fluorescence in situ hybridization (FISH) for *PAX3* (*PAX3* Break Apart FISH Probe Kit; CytoTest Inc., Rockville, MD, USA) and FISH for *SS18* (*SS18* Break Apart FISH Probe Kit; CytoTest Inc., Rockville, MD, USA). FISH was performed on interphase nuclei according to the manufacturer’s instructions. The results were considered positive when more than 30% of tumor nuclei had evidence of rearrangement in at least 100 tumor cells. The split signal was counted when the space between the two signals was larger than that of one signal.

## 3. Results

### 3.1. Clinicopathological Characteristics

From all analyzed cases of solid tumors located in the sinonasal region, 6 were identified as BSNS. Among them, three cases, which have been diagnosed before 2012 (cellular schwannoma, low-grade malignant peripheral nerve sheath tumor (MPNST), MPNST with heterologous rhabdomyoblastic differentiation) were reclassified. The mean age of patients was 63 years (range: 53 to 68 years old) with female predilection (4:2). More detailed clinical characteristics are described in Table 2.

**Figure 1:**
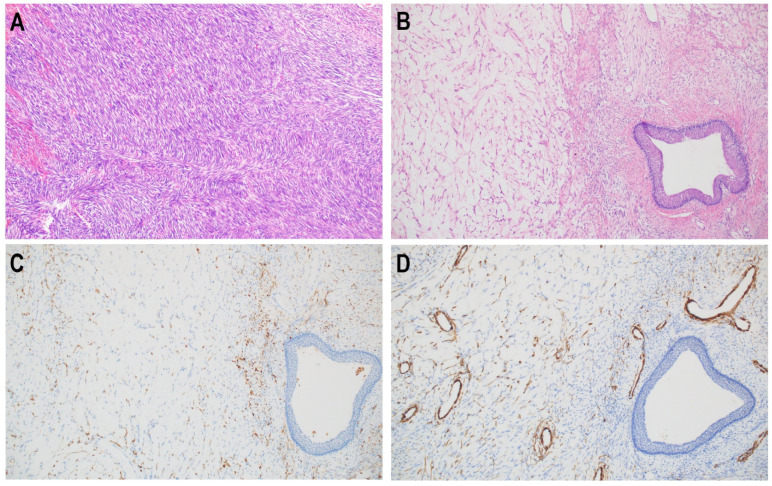
Low-power appearance of BSNS. Predominantly BSNS is a highly cellular, monotonous, bland-appearing spindle cell proliferation, arranged in medium to long fascicles often with prominent herringbone architecture (**A**). One case presented different morphology with prominent myxoid features (**B**). Myxoid component also showed expression of S100 (**C**) and SMA (**D**).

**Figure 2:**
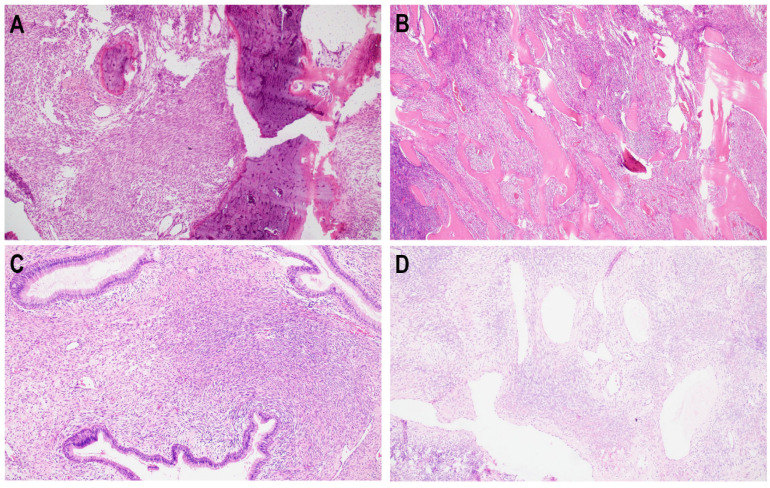
BSNS distinctive characteristic feature of BSNS is bone trabeculae infiltration, which was observed both, in incisional biopsy (**A**) and resection specimens (**B**). Other common findings are entrapped invaginations of hyperplastic respiratory epithelium (**C**) or stroma with “hemangiopericytoma-like” staghorn vessels (**D**).

The average diameter of BSNS biopsy samples was 0.8 cm, which accounted for 32% of the H&E stained slide average diameter of the subsequently resected tumor. The average size of the resection specimen was 2.5 cm. Histologically, all biopsy specimens presented as a monotonous, hypercellular proliferation of elongated, bland spindle cells arranged in medium to long fascicles partly forming a herringbone pattern (Figure 1A). The background stroma contained “hemangiopericytoma-like” staghorn vessels (Figure 2D). In one case prominent myxoid morphology was prevalent (Figure 1B). Characteristically, in the majority of cases (4/6), entrapped benign glands lined by proliferations of respiratory epithelium (Figure 2C) were observed. Bone trabeculae infiltration was present in three cases (Figure 2A,B) (Table 2). Mitotic activity was low, less than 1 per 10 high power fields and necrosis was absent. The morphology presented in small biopsy samples was representative of resection specimens in all analyzed BSNS cases.

### 3.2. Immunohistochemistry and FISH 

**Figure 3:**
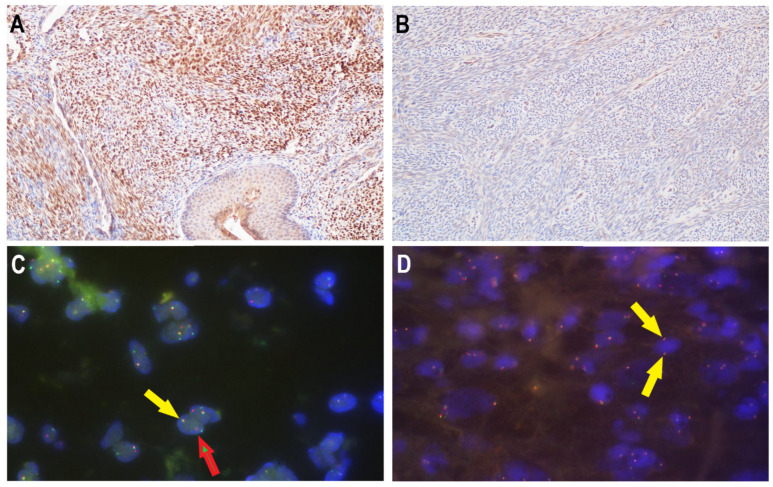
*PAX3* rearrangement. PAX3 expression IHC (**A**,**B**). Diffuse, strong nuclear staining was seen and the expression was distinctly stronger than the surrounding background (**A**). In PAX3 negative cases, only no specific background expression was observed (**B**). *PAX3* rearrangement by FISH (**C**,**D**). The yellow arrows indicate signals of an intact *PAX3* gene region and the separate red and green signals (red/green arrow) represent positive nuclei with PAX3 gene break (**C**).

In BSNS biopsy specimens, the co-expression of two hallmark proteins such as S100 and SMA was found in above 80% (5/6) of analyzed cases. One case was S100 positive but there was no expression of SMA. S0X10 such as other markers like h-caldesmon, STAT6, β-catenin, CD34, and CKAE1/3 were negative in all evaluated BSNS samples. The expression of H3K27me3 was retained (6/6). Desmin, as well as myogenin, was focally positive in one case (no. 6), which was previously misdiagnosed as MPNST with heterologous rhabdomyoblastic differentiation. However, the biopsy sample from the same patient was negative for desmin and another myogenic marker, MyoD1. MyoD1 expression was not seen in the remaining case confirming the lack of rhabdomyoblastic differentiation in cases 1–5. PAX3 expression by immunohistochemistry was demonstrated in all BSNS samples. Diffuse, strong nuclear staining was observed and the expression was distinctly stronger than the surrounding background (Figure 3).

The pattern of IHC expression was identical for paired biopsy and resection samples apart from one BSNS case (case 4), where diverging results of SMA expression occurred. All resection BSNS specimens were positive for SMA. Analysis of IHC patterns shows that SMA staining varied from diffuse (1 case) to focal (4 cases) and patchy (1 case), whereas S100 demonstrated focally (5 cases) or diffuse (1 case) positivity.

BSNS diagnosis was confirmed by *PAX3* rearrangement by FISH in five specimens. In four analyzed cases, FISH for *SS18* rearrangement was done and was negative, including the case of TLE-1 positive BSNS. Ancillary test results are described in Table 3.

## 4. Discussion

Soft tissue neoplasms of the head and neck region account for 15% of all sarcomas in adults and approximately 10% of them localize in the sinonasal tract. This region is characterized by a large diversity of entities- nearly all types of soft tissue tumors have been reported in the sinonasal tract so far [12,13,14]. Due to this fact, an accurate diagnosis, which is crucial for the prognosis as well as the treatment, could be challenging. Among the head and neck neoplasms, BSNS is considered a rare entity, with little over one hundred cases reported in the literature [1,2,3,5,12,15,16,17,18,19,20,21]. The first description of the entity was made by Lewis in 2012 [2]. Previously these tumors were misdiagnosed as other mesenchymal neoplasms such as LG MPNST, fibrosarcoma/unclassified spindle cell sarcoma or cellular schwannoma [2,10]. We also found three cases diagnosed before 2012 as MPNST or cellular schwannoma, which after careful evaluation were reclassified as BSNS. All cases presented morphological as well as immunophenotypic characteristics for BSNS.

Despite the fact that this entity has been present in the literature for 10 years, it is still referred to as a rare neoplasm and the diagnosis could pose some challenges. Especially small samples seem to be difficult to interpret, as the immunohistochemical markers could be expressed only focally. Nowadays, the biopsy of the suspicious lesion in the sinonasal tract area is the first step in the oncological diagnostic process. In our study, we sought to verify if some particular histological and immunohistochemical features and characteristics of BSNS are reflected in the incisional biopsy samples. 

Clinically BSNS presents distinct female predominance (female to male ratio of 2:1) and in the majority of cases occurs in patients in the fifth decade of life (range from 24 to 87 years) [6], which is reflected in our results. BSNS typically arises from the upper nasal cavity and ethmoid sinus. Other sinuses are less often affected [1]. In our series involvement of the nasal cavity, ethmoid sinuses and also other sinuses, skull base or orbit was present. 

However, a variety of sinonasal neoplasms may pathologically mimic BSNS, histological presentation of this entity shows some particular pathological features, which can be helpful in the distinction. The most common mark seen in 70% of cases is the presence of entrapment of hyperplastic respiratory epithelium, which leads to gland or cyst formation (so-called “pseudo-gland formation”). Lewis described this finding as unique to BSNS and absent in other mesenchymal tumors arising in the sinonasal tract [2]. We also observed this feature in almost all incisional biopsy specimens. In one case epithelium was not present in the sample (Table 2). Moreover, BSNS tumors commonly involve the adjacent bone, especially the orbit [22,23]. It may be reflected in the infiltration of bone trabeculae observed in the HE specimen and could be a helpful clue in the evaluation of a small biopsy sample. Other tumors, such as cellular schwannomas, could imitate bone infiltration, but typically they are well-circumscribed and have a pushing front into the bone [24]. In our series, the bone invasion was present in three samples, despite the absence of orbital destruction in computed tomography in one of them (Table 2). Nevertheless, we found both features: the presence of “pseudo-gland formation” and bone invasion helpful clues to the diagnosis of BSNS from incisional biopsy samples.

We also observed prominent myxoid morphology in one case of BSNS, which has not been reported so far. It is important to be aware of this pattern, as mimickers could also present myxoid areas. Myxoid change is a well-recognized feature of SS [25]; however, we confirmed that our sample harbored *PAX3* rearrangement and excluded *SS18* rearrangement. The features such as uniform spindle cell proliferation showing classic herringbone patterns, and overlapping cells should raise a suspicion of BSNS; however, they are not specific to the entity [1,4]. The presence of hemangiopericytoma-like staghorn vessels is characteristic of both BSNS and sinonasal SNGPC. Spindle cell neoplasms from a spectrum of low-grade tumors include benign (leiomyoma and schwannoma), borderline (sinonasal glomangiopericytoma (SNGPC) and solitary fibrous tumor (SFT)) and malignant (fibrosarcoma/unclassified spindle cell sarcoma, leiomyosarcoma, MPNST, BSNS and SS) tumors [10]. Some of these entities, such as SS, leiomyosarcoma or MPNST could demonstrate high-grade morphology as well. In some cases of BSNS, a few large cells with eccentric nuclei, prominent nucleoli and cells with bright, fibrillary eosinophilic cytoplasm and focal cross-striations suggestive of rhabdomyoblastic differentiation are present. In our series, we found only one case of myogenic differentiation confirmed in surgical specimens by myogenin expression. However, the biopsy sample from the same tumor did not show any features of rhabdomyoblastic differentiation. We think that the reason for that is limited tissue material and that the myogenin staining was present only focally in a few sarcoma cells. With regard to muscle markers, MyoD1 is considered a more specific marker for BSNS compared to myogenin. In the study of Loarer et al. myogenin was positive in 20% and MyoD1 in 91% [17]. In contrast, Wang’s study showed positivity for MyoD1 in only 4 of 25 cases [3]. The diversity of the MyoD1 profile could be explained by different antibodies used in the studies (clone 5.8A in Wang et al. and our studies and clone EP212 in Loarer et al. study). The positivity for muscle markers should always raise suspicion of spindle cell rhabdomyosarcoma. However, low-grade morphology, characteristic immunoprofile and molecular findings exclude this diagnosis. 

Although some of these pathological characteristics of BSNS are distinct, none are exclusive to this entity. The diagnosis of BSNS based on histological features alone is not possible due to the potential for pathological overlap. Therefore, immunophenotyping is a prerequisite for the diagnosis. Our study shows that biopsy and resection specimens of BSNS could be comparably useful in both histologic and immunohistochemical diagnostics. All BSNS cases we described showed typical immunophenotypes in resection specimens and were positive for S100 and SMA, while negative for SOX10. In one case SMA was negative in a biopsy specimen, while in a resection specimen it revealed a patchy pattern. The literature data revealed constant positivity for S100 and negativity for SOX10 in BSNS cases. SMA marker is variable and is positive in most cases [1,6,8]. We also confirmed the usefulness of the 3 markers mentioned in small biopsy samples; however, the absence of SAM expression should not exclude the diagnosis of BSNS. This lack of SMA expression in incisional biopsy samples could direct the diagnosis to tumors of neural origin, such as MPNST or schwannoma, which both express S100 and SOX10 and are typically negative for myogenic markers. Cellular schwannoma shows the diffuse expression of S100, while BSNS commonly presents focal patterns. Additionally, the SOX10 results are divergent. The second differential, MPNST could be only focally positive for S100. However, the other markers such as SOX10 or H3K27me3 were found to be more specific for MPNST and constantly negative in BSNS [24,26]. According to Carter et al., many of LG-MPNSTs reported in the sinonasal region actually represent BSNS [6]. 

The other pitfall could be triple-negative tumors, which require additional immunostainings such as STAT6 and CD34. The expression of these proteins confirms the diagnosis of SFT [27]. 

The group of smooth muscle tumors (SMT) may also arise in the differential diagnosis. They are characterized by SMA as well as desmin and h-caldesmon expressions. However, a very small number of SMT could exhibit S100 expression [26]. Moreover, in tumors with the following immunoprofile: S100+, SOX10-, SMA+ (diffuse reactivity) and the possibility of SMT should be taken into consideration and the confirmation of this diagnosis by expression of smooth muscle tumor markers is required. In cases without the expression of desmin or h-caldesmon, the evaluation of β-catenin should be done. A positive nuclear β-catenin reactivity in combination with diffuse SMA expression is highly characteristic of SNGPC [27]. This borderline tumor also shows a strong reaction with cyclin-D1, which can be used as an additional marker for the confirmation of the diagnosis [10,27]. The β-catenin immunoexpression results in BSNS are divergent. Reports of β-catenin positivity are limited to a few publications. A study by Rooper et al. is the only one that demonstrated almost constant nuclear positivity in this tumor type [19]. Another study reports that only a quarter (27%) of these cases showed focal or diffuse nuclear positivity [17]. In our series, no positivity for β-catenin in BSNS was observed and we support the statement that β-catenin IHC has a limited role as a diagnostic marker for BSNS [20,28]. 

Despite that S100(+), SOX(-), and SMA(+) immunoprofile is convincing for BSNS diagnosis, it is not always sufficient in distinguishing from all histologic mimics, such as monophasic SS, which could share the same immunoprofile. Although SS may demonstrate high-grade morphology as well, it should also be considered in the differential diagnosis. Up to 30% of SS could express S100 protein, which with particular morphological patterns could be misinterpreted as BSNS, especially in small biopsy samples [29]. Contrary to BSNS, SS shows epithelial differentiation detected by the expression of keratin or EMA. Despite EMA and TLE1 expressions being typical of SS [30], they could also be positive in BSNS (EMA, 63%; TLE1, 50%) [18]. We observed constant negativity for EMA and CKAE1/AE3 in all cases and diffuse positivity for TLE1 in one case, both in resection and biopsy samples. Moreover, SS harbors *SS18* gene rearrangement, which could be detected by the FISH break-apart probe. The mutation was not detected in BSNS and we confirm this statement in our study. However, the other molecular alteration, which is *PAX3* rearrangement, is characteristic of BSNS and the diagnosis often requires confirmation of its occurrence. PAX3 is a transcription factor controlling the differentiation of melanocytic, neural and myogenic cell lineage and has emerged as a potential oncogene in several different neoplasms, among them BSNS [3,31]. The *PAX3* gene is involved in recurrent chromosomal rearrangements, resulting in the creation of chimeric gene fusions most frequent with the *MAML3* fusion partner. Other molecular alterations include *PAX3-NCOA1* and *PAX3-FOXO1* fusions [15,16]. Diagnosis can be made either by genetics or immunohistochemical methods; however, both are not widely accessible. Jo et al. revealed that immunohistochemical expression of PAX3 can distinguish BSNS from its morphologic mimics with high sensitivity (100%) and specificity (98%) [18]. In our study, polyclonal PAX3 was used and we found the dilution 1:300 appropriate for diagnostics. However, besides characteristic nuclear expression, we also observed nonspecific background staining. Evaluation of the polyclonal PAX3 expression pattern could be challenging and it seems that FISH should be a standard method of confirming questionable cases and be routinely used in diagnostic practice. The most important limitation of FISH could be the age of FFPE specimens. In one examined case (above 10 years from tissue sampling) FISH failed to prove *PAX3* rearrangement. However, PAX3 expression was positive and in the context of histological and immunohistochemical results we established the diagnosis of BSNS. 

The findings of this study have to be seen in the light of some limitations. The sample size is small, so the conclusions should be made with caution. However, the BSNS is considered a rare entity and we did not find any study comparing the incisional biopsy and resection specimens in the context of histological and immunohistochemical features, including PAX3 immunohistochemical expression. 

## 5. Conclusions

In conclusion, from the authors’ experience, the incisional biopsy is a sufficient method to establish BSNS diagnosis in most cases. Morphological features such as entrapped benign glands lined by proliferations of the respiratory epithelium as well as bone trabeculae infiltration are important clues for the diagnosis of BSNS from small biopsy samples. We found S100, SOX10 and SMA immunostains useful as the screening markers when BSNS is suspected. In cases of divergent morphology and immunoprofile evaluation of *PAX3* rearrangement, either by FISH or IHC is vital for final diagnosis. Other immunostains such as β-catenin, CD34 or H3K27me3 could be helpful in differential diagnosis; however, FISH should be a standard method of confirming questionable cases and be routinely used in diagnostic practice. The authors stand that when BSNS is suspected due to its histological features, the first step should be performing S100, SOX10 and SMA immunostains and then, the next step should be molecular testing of *PAX3* rearrangement. The spectra of sinonasal low-grade spindle cell neoplasms are a wide and careful evaluation of the morphology, immunoprofile and molecular findings are essential for the diagnosis of BSNS.

## Figures and Tables

**Table 1:** Immunohistochemical staining protocols.

Stain	Clone	Dilution	Company	Platform	Address
S100	Poly	ready to use	Dako	Dako O/AS	Carpinteria, CA, USA
SMA	1A4 clone	ready to use	Dako	Dako AS	Carpinteria, CA, USA
SOX10	EP267 clone	ready to use	Roche	Roche	Branchburg, NJ, USA
desmin	D33 clone	ready to use	Dako	Dako O/AS	Carpinteria, CA, USA
h-caldesmon	h-CD colne	ready to use	Dako	Dako O/AS	Carpinteria, CA, USA
MyoD1	5.8A	1:100	Dako	Dako AS	Carpinteria, CA, USA
myogenin	F5D	ready to use	Dako	Dako O/AS	Carpinteria, CA, USA
b-catenin	B-Catenin-1 clone	ready to use	Dako	Dako AS	Carpinteria, CA, USA
STAT6	EP325 clone	1:50	Cell Marque	manual	Rocklin, CA, USA
CD34	QBEnd10	ready to use	Dako	Dako O/AS	Carpinteria, CA, USA
CKAE1/AE3	AE1/AE3 colne	ready to use	Dako	Dako O/AS	Carpinteria, CA, USA
EMA	E29 clone	ready to use	Dako	Dako AS	Carpinteria, CA, USA
TLE1	1F5 clone	1:100	Cell Marque	manual	Rocklin, CA, USA
PAX3	Polyclonal	1:400	Thermo Fisher Scientific	manual	Waltham, MA USA
H3K27me3	Monoclonal	1:2000	Cell Signaling Technology	manual	Danvers, MA, USA

**Table 2:** Clinicopathological characteristics of patients with BSNS.

No.	Age [Years]	Sex	Tumor Location	Orbital Destruction	Bone Infiltration	Respiratory Epithelium Invagination	Histological Pattern	pTNM	Treatment	Recurrence	Follow-Up [Months]
1	68	F	Nasal cavity, right maxillary sinus, bilateral sphenoid sinuses, bilateral ethmoid sinuses, nasopharynx	+	Bone not present in the specimen	+	Fascicular + myxoid	pT4N0M0	Surgery + adjuvant RTH	-	41
2	62	F	Nasal cavity, right ethmoid sinuses, skull base	+	+	-	Fascicular	pT4N0M0	Surgery + adjuvant RTH	-	40
3	62	F	Right ethmoid sinuses, right sphenoid sinus, right maxillary sinus	+	Bone not present in the specimen	Epithelium not present in the specimen	Fascicular	pT4N0M0	Surgery + adjuvant RTH	-	76
4	65	F	Right ethmoid sinuses, right sphenoid sinus, right maxillary sinus	-	+	+	Fascicular	pT4N0M0	Surgery	NA	NA
5	66	M	Nasal cavity, left ethmoid sinuses, left maxillary sinus	+	Bone not present in the specimen	+	Fascicular	pT4N0M0	Surgery + adjuvant RTH	+	21
6	53	M	Nasal cavity, right maxillary sinus, right and left ethmoid sinuses	+	+	+	Fascicular	pT4N0M0	Surgery + adjuvant RTH	+	153

**Table 3:** IHC and FISH findings in incisional biopsy and resection specimens.

No.		1 BS/RS	2 BS/RS	3 BS/RS	4 BS/RS	5 BS/RS	6 BS/RS
IHC	S100	(+) focal/(+) focal	(+) focal/(+) focal	(+) diffuse/(+) diffuse	(+) focal/(+)	(+) focal/NA	(+) focal/(+) focal
	SOX10	(-)/(-)	(-)/(-)	(-)/(-)	NA	(-)/NA	(-)/(-)
	SMA	(+) focal/(+) focal	(+) focal/(+) focal	(+) focal/(+) focal	(-)/(+) patchy	(+) diffuse/NA	(+) focal/(+) focal
	desmin	(-)/(-)	(-)/(-)	(-)/(-)	(-)/(-)	(-)/NA	(-)/(+) focal
	h-caldesmon	(-)/(-)	(-)/(-)	(-)/(-)	(-)/(-)	(-)/NA	(-)/(-)
	MyoD1	(-)/(-)	(-)/(-)	(-)/(-)	NA	(-)/NA	(-)/NA
	myogenin	NA	NA	NA	NA	NA	NA/(+) focal
	STAT6	(-)/(-)	(-)/(-)	NA	NA	(-)/NA	(-)/NA
	b-catenin	(-)/(-)	(-)/(-)	NA	NA	(-)/NA	(-)/NA
	CD34	(-)/(-)	(-)/(-)	(-)/(-)	(-)/(-)	(-)/NA	(-)/(-)
	CKAE1/3	(-)/(-)	(-)/(-)	(-)/(-)	(-)/(-)	(-)/NA	(-)/(-)
	EMA	(-)/(-)	(-)/(-)	(-)/(-)	(-)/(-)	(-)/NA	NA
	TLE-1	NA	NA	(+)/(+)	NA	NA	(-)/(-)
	H3K27m3	(+)/(+)	(+)/(+)	(+)/(+)	(+)/(+)	(+)/NA	NA/(+)
	PAX3	(+)/(+)	(+)/(+)	(+)/(+)	(+)/(+)	(+)/NA	NA/(+)
FISH	*PAX3*	(+)	(+)	(+)	ND	(+)	(+)
	*SS18*	(-)	(-)	(-)	NA	(-)	NA
Diagnosis		BSNS	BSNS	BSNS	BSNS	BSNS	BSNS

Abbreviations: BS—biopsy specimen; RS—resection specimen; NA—not available; ND—non-diagnostic.

## Data Availability

Not applicable.

## References

[1] Gross J., Fritchie K. (2020). Soft Tissue Special Issue: Biphenotypic Sinonasal Sarcoma: A Review with Emphasis on Differential Diagnosis. Head Neck Pathol..

[2] Lewis J.T., Oliveira A.M., Nascimento A.G., Schembri-Wismayer D., Moore E.A., Olsen K.D., Garcia J.G., Lonzo M.L., Lewis J.E. (2012). Low-grade Sinonasal Sarcoma With Neural and Myogenic Features. Am. J. Surg. Pathol..

[3] Wang X., Bledsoe K.L., Graham R., Asmann Y.W., Viswanatha D.S., Lewis J.E., Lewis J.T., Chou M.M., Yaszemski M.J., Jen J. (2014). Recurrent PAX3-MAML3 fusion in biphenotypic sinonasal sarcoma. Nat. Genet..

[4] Bishop J. (2016). Newly Described Tumor Entities in Sinonasal Tract Pathology. Head Neck Pathol..

[5] Powers K.A., Han L.M., Chiu A.G., Aly F.Z. (2015). Low-grade sinonasal sarcoma with neural and myogenic features—Diagnostic challenge and pathogenic insight. Oral Surg. Oral Med. Oral Pathol. Oral Radiol..

[6] Carter C., East E., McHugh J. (2018). Biphenotypic Sinonasal Sarcoma: A Review and Update. Arch. Pathol. Lab. Med..

[7] Llorente J.L., López F., Suárez C., Hermsen M.A. (2014). Sinonasal carcinoma: Clinical, pathological, genetic and therapeutic advances. Nat. Rev. Clin. Oncol..

[8] Martin E., Radomski S., Harley E. (2019). Sarcomas of the paranasal sinuses: An analysis of the SEER database. Laryngoscope Investig. Otolaryngol..

[9] El-Naggar A.K., Chan J.K.C., Grandis J.R., Takata T., Slootweg P.J. (2017). WHO Classification of Head and Neck Tumours.

[10] Thompson L., Franchi A. (2017). New tumor entities in the 4th edition of the World Health Organization classification of head and neck tumors: Nasal cavity, paranasal sinuses and skull base. Virchows Arch..

[11] Kak I., Perez-Ordoñez B. (2019). Sinonasal tract pathology: An updated review of select entities. Diagn. Histopathol..

[12] Andreasen S., Bishop J.A., Hellquist H., Hunt J., Kiss K., Rinaldo A., Skálová A., Willems S.M., Williams M., Ferlito A. (2018). Biphenotypic sinonasal sarcoma: Demographics, clinicopathological characteristics, molecular features, and prognosis of a recently described entity. Virchows Arch..

[13] Szablewski V., Neuville A., Terrier P., Laé M., Schaub R., Garrel R., Coindre J.-M., Costes V. (2014). Adult sinonasal soft tissue sarcoma: Analysis of 48 cases from the French Sarcoma Group database. Laryngoscope.

[14] Jo V., Fletcher C. (2014). WHO classification of soft tissue tumours: An update based on the 2013 (4th) edition. Pathology.

[15] Wong W.J., Lauria A., Hornick J.L., Xiao S., Fletcher J.A., Marino-Enriquez A. (2015). Alternate PAX3-FOXO1 oncogenic fusion in biphenotypic sinonasal sarcoma. Genes Chromosomes Cancer.

[16] Huang S.C., Ghossein R.A., Bishop J.A., Zhang L., Chen T.C., Huang H.Y., Antonescu C.R. (2016). Novel PAX3-NCOA1 Fusions in Biphenotypic Sinonasal Sarcoma With Focal Rhabdomyoblastic Differentiation. Am. J. Surg. Pathol..

[17] Le Loarer F., Laffont S., Lesluyes T., Tirode F., Antonescu C., Baglin A.C., Delespaul L., Soubeyran I., Hostein I., Pérot G. (2019). Clinicopathologic and Molecular Features of a Series of 41 Biphenotypic Sinonasal Sarcomas Expanding Their Molecular Spectrum. Am. J. Surg. Pathol..

[18] Jo V.Y., Mariño-Enríquez A., Fletcher C.D., Hornick J.L. (2018). Expression of PAX3 Distinguishes Biphenotypic Sinonasal Sarcoma From Histologic Mimics. Am. J. Surg. Pathol..

[19] Rooper L.M., Huang S.-C., Antonescu C.R., Westra W.H., Bishop J.A. (2016). Biphenotypic sinonasal sarcoma: An expanded immunoprofile including consistent nuclear β-catenin positivity and absence of SOX10 expression. Hum. Pathol..

[20] Kakkar A., Rajeshwari M., Sakthivel P., Sharma M.C., Sharma S.C. (2018). Biphenotypic sinonasal sarcoma: A series of six cases with evaluation of role of β-catenin immunohistochemistry in differential diagnosis. Ann. Diagn. Pathol..

[21] Fritchie K.J., Jin L., Wang X., Graham R., Torbenson M.S., E Lewis J., Rivera M., Garcia J.J., Schembri-Wismayer D.J., Westendorf J.J. (2016). Fusion gene profile of biphenotypic sinonasal sarcoma: An analysis of 44 cases. Histopathology.

[22] Miglani A., Lal D., Weindling S.M., Wood C.P., Hoxworth J.M. (2019). Imaging Characteristics and Clinical Outcomes of Biphenotypic Sinonasal Sarcoma. Laryngoscope Investig. Otolaryngol..

[23] Lin Y., Liao B., Han A. (2017). Biphenotypic sinonasal sarcoma with diffuse infiltration and intracranial extension: A case report. Int. J. Clin. Exp. Pathol..

[24] White W., Shiu M.H., Rosenblum M.K., Erlandson R.A., Woodruff J.M. (1990). Cellular schwannoma. A clinicopathologic study of 57 patients and 58 tumors. Cancer.

[25] Choi J.H., Shin D.S., Bae Y.K., Kim M.J., Shim Y.R., Jung J.Y. (2005). Synovial Sarcoma with Massive Myxoid Feature: A Case Report. J. Pathol. Transl. Med..

[26] Karamchandani J.R., Nielsen T.O., van de Rijn M., West R.B. (2012). Sox10 and S100 in the Diagnosis of Soft-tissue Neoplasms. Appl. Immunohistochem. Mol. Morphol..

[27] Thompson L., Fanburg-Smith J. (2016). Update on Select Benign Mesenchymal and Meningothelial Sinonasal Tract Lesions. Head Neck Pathol..

[28] Jo V., Fletcher C. (2016). Nuclear β-Catenin Expression is Frequent in Sinonasal Hemangiopericytoma and Its Mimics. Head Neck Pathol..

[29] Hoda S. (2014). Enzinger and Weiss’s Soft Tissue Tumors, 6th Edition. Adv. Anat. Pathol..

[30] Foo W.C., Cruise M., Wick M., Hornick J. (2011). Immunohistochemical Staining for TLE1 Distinguishes Synovial Sarcoma From Histologic Mimics. Am. J. Clin. Pathol..

[31] Wang Q., Fang W.H., Krupinski J., Kumar S., Slevin M., Kumar P. (2008). Pax genes in embryogenesis and oncogenesis. J. Cell. Mol. Med..

